# Pathomorphological Features of Lung Damage Caused by Infection with Various Variants of SARS-CoV-2 in Humanized Model Animals

**DOI:** 10.1134/S1607672925601787

**Published:** 2026-02-04

**Authors:** A. S. Chernov, V. A. Kazakov, I. S. Gogleva, F. A. Mescheryakov, A. A. Kudriaeva, A. P. Bogachuk, I. V. Smirnov, G. B. Telegin, A. A. Belogurov, A. G. Gabibov

**Affiliations:** 1 Shemyakin–Ovchinnikov Institute of Bioorganic Chemistry of the Russian Academy of Sciences, Moscow, Russian Federation; 2 Russian University of Medicine, Ministry of Health of the Russian Federation, Moscow, Russia

**Keywords:** SARS-CoV-2, viral variants, humanized C57BL/6 hAEC2-TG+ mice, experimental modeling of COVID-19

## Abstract

The genotypic variability of the SARS-CoV-2 virus is extremely high, and the emergence of new strains raises concerns about their possible high virulence and ability to bypass responses of the body’s immune system induced by previous infection or vaccination. Therefore, one of the main tasks is to study the pathogenesis of various variants of the virus using experimental animal biomodels of SARS-CoV-2 to quickly find methods and approaches to fighting new viruses. The study was performed on humanized mice of the C57BL/6-Tgtn line. Mice were infected intranasally at different doses with three variants of the SARS-CoV-2 virus. We showed that humanized hACE2 mice, when infected with all three variants of the SARS-CoV-2 virus, showed typical pathological changes in lung consistency comparable to those found in COVID-19 in humans. At a dose of 4 log plaque-forming unit (PFU), all variants showed 100% mortality. In a comparative assessment of different variants of the SARS-CoV-2 virus in hACE2 humanized mouse model, it was found that the Delta variant leads to more severe damage compared to Wuhan or Omicron.

## INTRODUCTION

Since the SARS-CoV-2 virus first emerged in Wuhan, China, in late 2019 [1], its genetic material has undergone numerous changes, some of which have affected its transmissibility, the severity of the disease it causes, and the effectiveness of vaccines, diagnostics, and treatments used against it [2]. SARS-CoV-2 virus genomes that differ from each other only in certain fragments of the genetic sequence are called variants [3]. Currently, all SARS-CoV-2 coronavirus forms are variants.

By mid-summer 2021, the Delta variant was prevalent in most countries, including Russia: of the 244 virus genomes sequenced, 242 were Delta variants. One of the key mutations that gives Delta an advantage over other variants is a mutation in the protein region where furin cleavage occurs [4]. This mutation increases the proportion of activated viral particles produced after replication by 25% compared to the original variant [5], making it 40–60% more infectious [6]. Its contagiousness is similar to that of chickenpox.

The Omicron variant was first identified on November 9, 2021, in Botswana and a few days later in South Africa [7]. According to the WHO, the new variant spread more rapidly than previous ones, as evidenced by data from Russian specialists [8]. This fact can be explained by the high rate of virus reproduction in the bronchi and nasal epithelium, as well as its ability to bypass the protective functions of the immune system.

Animal biomodels that reproduce the clinical and pathological features of COVID-19 in humans are an important tool for studying the pathogenesis, mechanisms, and transmission routes of the pathogen. Currently, there are several transgenic mouse models in which hACE2 expression is under the control of a tissue-specific promoter: K-18-hACE2 mice with the human K-18 promoter [9]; HFH4-ACE2 mice with the human HFH4 promoter [10]; rodents with the ACE2 promoter [15]; and AC70, AC22 and AC63 mice with the CAG promoter [12]. Of all the presented models, the CAG-hACE2 transgenic mice were found to be most promising, since the hACE2 protein in them is expressed not only in the lungs, but also in the brain [13]. Brain infection is secondary to lung infection, where marked activation of inflammatory mediators is observed as early as 2 days after infection [13]. For these reasons, CAG-hACE2 mice are highly susceptible to SARS-CoV-2, with the lungs and brain being the primary targets during infection.

In view of the above, the aim of this study was to compare the pathomorphological features of lung damage in experimental animals when they were infected with different virus variants at different doses.

## MATERIALS AND METHODS

Three variants of the SARS-CoV-2 virus were used in the study: (1) the SARS-CoV-2 virus, variant hCoV-19/Russia/GAM-Omicron/2021 (genetic line B.1.1.529, Omicron); (2) the SARS-CoV-2 virus, variant hCoV-19/Russia/SPE-RІІ-32661V-2021 (genetic line B.1.617. (Indian variant) B. 1.617.3 according to the classification of May 12, 2021. Characteristic mutations GISAID EPI_ISI_1797437); and (3) the SARS-CoV-2 virus, variant B (line B.1.1, Wuhan).

In experiments, we used a continuous culture of African green monkey kidney cells, Vero Cl008, which was grown and maintained using Eagle’s Medium (MEM) with Hanks’ solution containing 7.5 and 2% fetal bovine serum, respectively.

The study involved 60 mice (females and males) of the CAG-hACE2-IRES-Luc-Tg line aged 8–10 weeks [14]. The animals were kept under standard conditions at the Laboratory Animal Breeding Facility of the Institute of Bioorganic Chemistry of the Russian Academy of Sciences (Development of the bioresource collection “Collection of laboratory rodents of SPF status for fundamental, biomedical, and pharmacological research of the Institute of Bioorganic Chemistry of the Russian Academy of Sciences” no. 075-15-2021-1067).

Mice were infected with the virus in a specialized laboratory (BSL-3 biosafety level) after a 3-day adaptation period. Animals were infected intranasally at doses of 1–4 log PFU in a total volume of 20 µL. The infected animals were observed for 12 days, with monitoring of their appearance, behavior, and appetite. In mice that died during the observation period and were euthanized on day 12, the level of SARS-CoV-2 virus accumulation in internal organs was assessed by the formation of negative colonies in Vero Cl008 cell culture.

The infectious activity of the virus was assessed in a Vero Cl008 cell culture by the formation of negative colonies under agar coating [15]. Cell suspension at a density of 200 000/mL was added to sterile plastic cell culture flasks and incubated for 24 h in a CO_2_ incubator in a 5% CO_2_ atmosphere at 37°C until a continuous monolayer formed. Flasks with a continuous monolayer were used to determine the infectious titer of the virus. At least two flasks were used for each tested dose of the drug and the control. Virus adsorption time was 60 min. Primary (secondary) agar coating was prepared using Earle’s glucose–saline solution containing L-glutamine, an amino acid vitamin complex, antibiotics, and fetal bovine serum. The infected cell monolayer was incubated for 3–5 days, the first coating was applied after 3 days of incubation, the second coating was applied 24 h later, and the negative colonies were counted on day 5 (for B and Delta variants) and on days 5–7 for the Omicron variant. Neutral Red solution was added to the secondary agar coating (6 mL per 100 mL of coating) instead of an equal volume of water. The biological activity (*A*) of the virus strain culture (in PFU/mL) was calculated using the formula:

1 $$A = \frac{{\bar {\bar {a}} \cdot {{b}_{n}}}}{c},$$

where $\bar {\bar {a}}$ is the average weighted number of plaques per flask, *b*_*n*_ is the degree of highest dilution, and *c* is the inoculum volume, mL.

2 $$\bar {\bar {a}} = \frac{{{{{\bar {a}}}_{1}} + {{{\bar {a}}}_{2}} + ... + {{{\bar {a}}}_{n}}}}{{{{b}_{n}}\left( {\frac{1}{{{{b}_{1}}}} + \frac{1}{{{{b}_{2}}}} + ... + \frac{1}{{{{b}_{n}}}}} \right)}},$$

where ${{\bar {a}}_{1}}$ … ${{\bar {a}}_{n}}$ is the average number of plaques in 1 to *n* dilution of the test material; ${{b}_{1}}$ … ${{b}_{n}}$ is the degree of dilution of the test material.

Lung samples were fixed in 10% neutral formalin for 2 weeks, after which one fragment from each lung lobe was excised. The samples were rinsed in running water, dehydrated in ascending concentrations of ethanol, and embedded in paraffin. Paraffin sections (4–5 µm thick), stained with hematoxylin and eosin, were examined using conventional light microscopy with an AxioScope.A1 microscope (Carl Zeiss, Germany). Photomicrographs of histological specimens were obtained using an Axiocam 305 color high-resolution camera (Carl Zeiss, Germany) and ZEN 2.6 lite software (Carl Zeiss, Germany). The severity of various inflammatory phenomena in the lungs was assessed by a semiquantitative method (in points) according to the following scale: 0—within normal limits, 1—weak, 2—moderate, and 3—severe.

The results were statistically processed using the Statistica software (StatSoft®, v.12.6, Tulsa, OK, United States). The results are presented as the mean value and standard deviation.

## RESULTS AND DISCUSSION

The susceptibility of hACE2 mice to SARS-CoV-2 variant B (Wuhan) was assessed at intranasal infection at doses of 1, 2, 3, and 4 log PFU (Table 1). Monitoring of the animals for 12 days revealed that the behavior, mobility, and appetite of infected animals did not differ from physiological norms. No obvious clinical symptoms were observed in any of the infected animals. Intranasal infection of hACE2 mice with doses of 3–4 log PFU resulted in 100% mortality. When mice were infected with doses of 1–2 log PFU, the survival rate was 60%. The virus was detected in the lung tissue of all dead mice (Table 1). When mice were infected with a dose of 3 log PFU, the viral load level in the mice that died on day 6 was 3.4 PFU/g, whereas on day 7 it was 2.6 PFU/g. The average virus accumulation rate in this group was 2.9 ± 0.2 PFU/g (Table 1). When LD_50_ was calculated using the Van der Waerden method, the value of this index was 178 ^x^/:3 PFU/mouse.

**Table 1.** Assessment of the viral load in the lungs of dead mice and the sensitivity of hACE2 mice to the SARS-CoV-2 virus, variant B (Wuhan) at intranasal infection

Infection dose, log PFU	Frequency of animal deaths	Animal death, %	Average life time before death, days	Level of virus accumulation in the lungs, PFU/g
4	5/5	100	4.0 ± 0	2.6 ± 0.3
3	5/5	100	4.0 ± 0	2.9 ± 0.2
2	2/5	40	9.8 ± 3.9	1.3 ± 0.2
1	2/5	40	11.3 ± 2.8	1.5 ± 0.2

When hACE2 mice were infected intranasally with the SARS-CoV-2 virus, variant B (Wuhan) at a dose of 3 log PFU, the level of accumulation of the pathogen on the 3rd day after infection (euthanasia) in the brain tissue was 5.12 log PFU/g, whereas in the lungs it was less than 1.0 log PFU/g (Table 2).

**Table 2.** Variant B, infection dose 3 log PFU, cervical dislocation

Days after infection	Brain	Lungs
1	0.0 ± 0.0	0.0 ± 0.0
2	0.0 ± 0.0	0.0 ± 0.0
3	5.12 log PFU/g	1 log PFU/g (the virus was found in 10% of the homogenate suspension)

In the animals that died on day 5, the level of virus accumulation in the brain was 6.8 log PFU/g, while in the lungs it was only approximately 2.0 log PFU/g (Table 3). Thus, virus accumulation in the brain tissue was significantly higher than in the target organ. Virus accumulation was also detected in the liver, spleen, and heart (Table 3).

**Table 3.** Accumulation of virus variant B (Wuhan) in different organs of infected mice

Organs	PFU/g
Brain	6.8 ± 0.03
Lung	2.0 ± 0.2
Liver	2.85 ± 0.06
Spleen	3.10 ± 0.14
Heart	1.87 ± 0.19
Kidneys	Not identified

The assessment of the hematological and biochemical parameters of the blood of healthy and hACE2-infected mice did not reveal any differences (Table 4).

**Table 4.** Hematological and biochemical parameters (in living) C57BL/6 hAEC2-TG+ mice on the 3rd day after infection with the SARS-CoV-2 virus, variant B (Wuhan) at a dose of 3 log PFU

Index	Infected	Intact
Red blood cells, 10^12^/L	8.4 ± 0.1	8.3 ± 0.1
Leukocytes, 10^9^/L	0.7 ± 0.1	0.9 ± 0.1
Lymphocytes, 10^9^/L	0.6 ± 0.0	0.8 ± 0.0
Granulocytes, 10^9^/L	0.2 ± 0.1	0.1 ± 0.0
Creatinine, μM/L	193 ± 2.0	223 ± 1.0
Urea, mM/L	0.97 ± 0.01	1.05 ± 0.10
AST, mM/h L	2.00 ± 0.15	2.58 ± 1.07
ALT, mM/h L	0.42 ± 0.30	0.29 ± 0.06
Creatinine kinase, U/L	2270.3 ± 1240.2	1037.3 ± 23.3
Lactate dehydrogenase, U/L	1997.3 ± 29.9	1946.0 ± 15.0

The viral load was also assessed in the lungs of dead hACE2 mice that were infected intranasally with the SARS-CoV-2 virus, variant hCoV-19/Russia/SPE-RІІ-32661V-2021 (Delta, line B.1.617.3, Indian variant). Intranasal infection with the SARS-CoV-2 virus at a dose of 3–4 log PFU resulted in 100% mortality; the average survival time before death was 5.8 days (Table 5).

**Table 5.** Assessment of the viral load in the lungs of dead mice and the sensitivity of hACE2 mice to the SARS-CoV-2 virus, variant hCoV-19/Russia/SPE-RIІ-32661V-2021 (Delta, line B.1.617.3, Indian variant) at intranasal infection

Infection dose, log PFU	Frequency of animal deaths	Animal death, %	Average life time before death, days	Level of virus accumulation in the lungs, PFU/g
4	5/5	100	5.6 ± 0.2	2.0 ± 0.3
3	5/5	100	5.4 ± 0.2	1.7 ± 1.0
2	3/5	60	5.3 ± 0.3	1.3 ± 1.0
1	3/5	60	6.7 ± 0.3	1.3 ± 1.0

When animals were infected with a dose of 1–2 log PFU, the mortality rate was 60%, the average survival time of mice until death was 6.7 and 5.3 days, respectively. When mice were infected with a dose of 4 log PFU, the level of virus accumulation in the lungs was 1.3 PFU/g in 40% and 2.6 PFU/g in 60% of animals. The average virus accumulation in the lungs of animals in this group was 2.0 ± 0.3 PFU/g. When mice were infected with a dose of 3 log PFU, the level of virus accumulation in the lungs was 1.3 PFU/g in 60% and 2.1 PFU/g in 40% of animals. The average virus accumulation in the lungs of animals in this group was 1.7 ± 0.6 PFU/g. When mice were infected with a dose of 1–2 PFU, the level of virus accumulation in the lungs was 100%. When LD_50_ was calculated using the Van der Waerden method, the value of this index was 79.56 ^x^/:2.7 PFU/mouse, 1.9 ± 0.4 PFU.

Mice were infected intranasally with the SARS-CoV-2 virus, variant hCoV-19/Russia/GAM-Omicron/2021 (line B.1.1.529, Omicron) at a dose of 1–4 log PFU in 20 μL per mouse. Monitoring of the animals’ condition showed that the behavior, mobility, and appetite of the infected animals did not deviate from the physiological norm. Intranasal infection of hACE2 mice with the SARS-CoV-2 virus, variant hCoV-19/Russia/GAM-Omicron/2021 (line B.1.1.529) at a dose of 4 log PFU resulted in 100% mortality; the average survival time until death was 7.6 days (Table 6). When infected with doses of 1 and 3 log PFU, 60% of the animals died; the average survival time until death was 7.6 and 9.3 days, respectively. When mice were infected with a dose of 2 PFU, the mortality rate was 80%, with the average survival time until death being 9.3 days.

**Table 6.** Assessment of the sensitivity of hACE2 mice to the SARS-CoV-2 virus, variant hCoV-19/Russia/GAM-Omicron/2021 (line B.1.1.529) at intranasal infection

Infection dose, log PFU	Frequency of animal deaths	Animal death, %	Average life time before death, days	Level of virus accumulation in the lungs, PFU/g
4	5/5	100	7.6 ± 0.2	3.3 ± 0.1
3	3/5	60	7.6 ± 0.3	3.3 ± 0.1
2	4/5	80	9.3 ± 0.3	2.9 ± 0.1
1	3/5	60	9.3 ± 0.9	2.3 ± 0.1

It should be noted that, at an infection dose of 1–2 log PFU, the animals that died on days 9, 10, and 11 after infection with the SARS-CoV-2 virus showed reduced mobility, heavy breathing, and wheezing in the last 3 days. We determined the viral load in the lungs of the dead hACE2 mice infected intranasally with the SARS-CoV-2 virus, variant hCoV-19/Russia/GAM-Omicron/2021 (line B.1.1.529). The data presented in Table 6 show that the level of virus accumulation in the target organ virtually did not depend of the infection dose. When the LD_50_ value was calculated using the van der Waerden method, it was 125.9^x^/:3.7 PFU/mouse, 2.1 ± 0.6 log PFU.

**Fig. 1.**
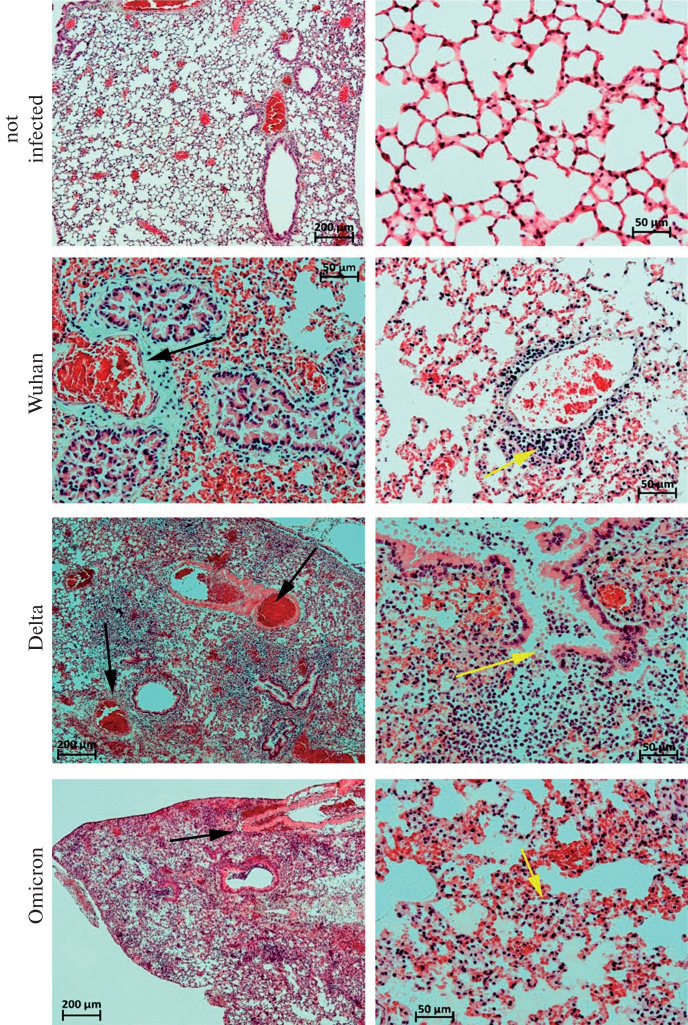
Lung fragments of intact hACE2 mice (a, b) and those infected with the Wuhan (c, d), Delta (e, f), and Omicron (g, h) variants of SARS-CoV-2, which died within 5–8 days after infection. Hematoxylin and eosin staining. Magnification, 50× (a, d, g) and 200× (b, c, d, e, h). (a, b) Lung fragment of an intact hACE2 mouse. (c, d) Lung fragments of an hACE2 mouse infected with the Wuhan variant of SARS-CoV-2 at a dose of 3 log PFU and died on day 7: pronounced disturbances at the level of the microcirculatory bed, desquamation of the respiratory epithelium, focal perivascular mononuclear infiltration. (d, e) Lung fragments of hACE mice infected with the Delta variant at doses of 3 (d) and 4 (e) log PFU and died on days 5 (d) and 6 (e) after infection: bronchial perforation with focal necrosis of the lobe. (g, h) Lung fragments of hACE mice infected with the Omicron variant at doses of 1 (g) and 3 (h) log PFU and died on day 8 after infection: bronchial perforation with focal necrosis of the lobe (g), hyaline-like membranes and transudate in the alveolar lumen (h).

In contrast to the healthy mice (Figs. 1a, 1b), the following pathomorphological picture was observed in the lungs of hACE2 mice infected with the Wuhan variant of SARS-CoV-2 at doses of 2–4 log PFU died on days 6–7: diffuse pronounced congestion of large vessels, as well as microcirculatory vessels with stasis and sludge of erythrocytes (Figs. 1c, 1d). Focal presence of a few macrophages, neutrophils, and lymphocytes was noted in the alveolar walls, with focal perivascular mononuclear infiltration detected around some vessels. Hyaline-like membranes and transudate were observed in the lumen of some alveoli in different lobes of the lungs. In the lumen of most bronchi, pronounced desquamation of the respiratory epithelium with initial destruction of the integrity of the bronchial wall was observed. The pathomorphological changes described above in the autopsy samples of the lungs of  hACE2 mice infected with the Wuhan strain of SARS-CoV-2 at doses of 2–4 log PFU were clearly dose-dependent.

Half of the hACE2 mice that were infected with the Delta variant at doses of 2–4 log PFU and died 5–6 days after infection typically had numerous large foci of necrosis in various lung lobes resulting from bronchial wall perforation (Figs. 1e, 1f). The necrotic foci were abundantly infiltrated with segmented neutrophils and numerous mononuclear cells. In the necrotic foci, the interalveolar septa were destroyed, and hyaline-like membranes and transudate could be seen in the lumens of intact alveoli. In cases where necrosis was not observed, the overall picture was similar to that observed in the mice infected with the Wuhan variant: pronounced disturbances in the microcirculation with erythrocyte stasis and sludge, hyaline-like membranes in the alveolar lumens, and massive transudate areas. Minor to moderate mononuclear cell infiltration was detected in the alveolar walls, and focal mononuclear cell infiltrates were observed in the perivascular space. In general, Delta is characterized by dose dependence in terms of the extent of lung tissue damage and the severity of the pathomorphological picture.

In the lungs of hACE2 mice that were infected with the Omicron strain at doses of 1–4 log PFU and died 7–9 days after infection, the pattern of pathological changes was generally similar to that observed in the case of infection with the Wuhan SARS-CoV-2 strain at a dose of 3 log PFU, with no apparent dose dependence. In all cases, significant disturbances in the microcirculation were observed, with signs of erythrocyte stasis and sludge (Figs. 1g, 1h). Hyaline-like membranes and massive areas of transudates were observed in the alveolar lumens. Moderate to severe mononuclear infiltration was observed in the alveolar walls, and focal mononuclear infiltrates (neutrophilic infiltrates in one case) were detected in the perivascular space. In one male rat infected with the Omicron strain at a dose of 1 log PFU, extensive foci of necrosis with destruction of the interalveolar septa and abundant tissue infiltration with segmented leukocytes were observed in two lung lobes. In both cases, bronchial perforation in the necrotic foci was observed. Given the above, no obvious dose-response relationship was observed for Omicron doses of 1–3 log PFU. At the high infection dose (4 log PFU), all animals with typical lung pathologies died. Survived hACE2 mice infected with different SARS-CoV-2 variants and euthanized on day 12 of the study did not show any obvious lung pathological changes.

Summary data on the frequency of occurrence of pathohistological signs in the lungs of hACE2 mice infected with the Wuhan strain of SARS-CoV-2 at a dose of 1–4 log PFU and the Omicron and Delta strains at doses of 1–4 log PFU are presented in Table 7.

**Table 7.** Frequency of occurrence of pathomorphological changes (in points) in the lungs of hACE2 mice infected with various variants of SARS-CoV-2, which died within 5–9 days after infection

Pathomorphological changes in the lungs of mice	Wuhan	Delta	Omicron
Infection dose, log PFU	1, 2, 3, 4	1, 2, 3, 4	1, 2, 3, 4
Dose dependence according to the pathohistological picture in the lungs	Yes	Yes	No
Microcirculation disorders—plethora, stasis, sludge of erythrocytes, microthrombosis	3	3	3
Hyaline-like membranes	2	2	2
Transudate in the lumen of the pulmonary acini	2	2	2
Infiltration of alveolar walls with mononuclear cells	1	1	1
Focal perivascular mononuclear cell infiltration	1	1	1
Desquamation of the respiratory epithelium of the bronchi	3	3	2
Foci of necrosis in the lungs as a result of perforation of the bronchial wall	0	3	1

As is currently known, mouse strains are not susceptible to the original SARS-CoV-2 variant that caused COVID-19. The key receptor for interaction with SARS-CoV-2 in humans is ACE2, which interacts with it through the receptor-binding domain (RBD) of the viral S protein. The mouse ACE2 receptor (mACE2) is not tropic to SARS-CoV-2, which was the basis for the use of a special humanized transgenic mouse strain, C57BL/6 hAEC2-TG+, expressing human ACE2 [16].

The virological and pathological characteristics obtained by us demonstrated that all virus variants effectively infected hACE2 mice, which is consistent with previous studies [16]. The use of the virus at doses of 3–4 log PFU resulted in 100% mortality in all infected hACE2 mice, demonstrating the high sensitivity and validity of the model used. These data make this model the preferred model for SARS-CoV-2 research, as opposed to, e.g., Syrian hamsters, which after infection with high doses of the virus (5–6 log PFU) develop only moderate signs of clinical disease and lung pathology [13, 17, 18].

Histological examination of lung autopsy specimens obtained from hACE2 mice infected with various SARS-CoV-2 variants demonstrated a typical pathomorphological picture in the target organ, characterized by pronounced diffuse microvascular congestion with erythrocyte stasis and sludge, signs of thrombosis, as well as infiltration of the alveolar walls with inflammatory cells and the presence of hyaline-like membranes and transudate in the pulmonary acini. In terms of a 50% probability of bronchial wall perforation, the most aggressive SARS-CoV-2 variant at intranasal infection of hACE2 mice is the Delta variant, which, along with the Wuhan variant, has demonstrated dose-dependent target organ damage.

It was shown for the Wuhan variant that the virus accumulation in the brain tissue is significantly higher than in the target organ. Possibly, in future studies, another way of infection should be considered, because intranasal administration provides the shortest route for the virus entry into the brain and its subsequent accelerated replication.

## CONCLUSIONS

C57BL/6 hAEC2-TG+ mice (C57BL/6- Tgtn(CAG- human AEC2-IRES-Luciferase-WPRE-polyA)) are highly susceptible to the SARS-CoV-2 variant B (Wuhan), hCoV-19/Russia/SPE-RІІ-32661V-2021 variant (Delta, B.1.617.3 line, Indian variant), and   hCoV-19/Russia/GAM-Omicron/2021 variant (B.1.1.529 line) in the case of intranasal infection. A comparative assessment of the pathogenicity of various SARS-CoV-2 virus variants in the humanized C57BL/6 hAEC2-TG+ mouse model showed that the Delta variant leads to more severe damage compared to the Wuhan and Omicron variants, which correlates well with clinical observations in humans and the results of our pathohistological examination of the lungs of infected mice [19]. The Omicron variant is similar to the Delta variant in terms of the severity of pathomorphological changes in the lungs, but does not have clear dose-dependent characteristics of the damaging effect on the target organ, unlike the Wuhan and Delta variants.
